# Analogous Foot-and-Mouth Disease1Read at Urbana, February 16, 1903, before the Illinois State Veterinary Medical Association.

**Published:** 1903-05

**Authors:** C. C. Mills

**Affiliations:** Decatur, Illinois


### ANALOGOUS FOOT-AND-MOUTH DISEASE.[1]

By C. C. Mills, V.S.,

DECATUR, ILLINOIS.

It is with no desire to enter into any exhaustive treatise on the disease I have in mind or to consume much time of this Association in considering a disease or symptoms which perhaps are only infrequently met with. It may be that the subject as announced does not comprehend the true nature of the affection in cattle which, to my mind, bears close analogy, in very many clinical symptoms at least, to the malady which recently has been the cause of so much concern in the New England States and toward the extermination of which the best efforts of the Bureau of Animal Industry have been directed. The symptoms, duration, and general character of this contagious foot-and-mouth disease is fresh in the minds of all reading veterinarians.

But there can be no doubt that the affection occasionally seen among cattle, and which is the subject of this paper, in common terms can justly claim the name of foot-and-mouth disease. In fact, these are the prominent symptoms, and yet I do not wish to be understood as claiming its proper classification as the contagious epizootic aphtha. Neither have I any new disease to herald, for doubtless every veterinarian in these parts has had occasion to prescribe for just such cases as I have in mind, and I hope some at least have been able to trace the true origin better than I, for it is with the

[1] Read at Urbana, February 16, 1903, before the Illinois State Veterinary Medical Association.

hope of receiving aid in the proper classification of this disease rather than to advance any new theory to the profession that I have suggested this subject.

I have been able to find but little in the professional literature which at all fits the case, and this naturally has caused me to wonder why? Within the past ten years, perhaps, I have seen but three or four seasons when any such symptoms have been found, and these outbreaks seemed to vary from being quite general at one time while at another only a few cases would be seen or heard of. The first outbreak which came under my notice was the most general and consequently caused the greatest loss. I have not the data at hand to be exact, but this was about ten years ago. In the late summer and early fall seasons fully half of the cattle over quite an extended area of country contracted the disease, which was characterized by a fever ranging from 103° to 105½° in the early stages and which continued for a period of from three or four days to a week. An accompanying soreness with weakness and perhaps a stupid or a humped-up appearance were usually present. Such animals eat but little or none at all when first noticed, and usually look gaunt when the owner or attendant's attention is first attracted. The dental or incisor pad becomes extremely sensitive as a usual thing, and in appearance is of a very dark or purple color, and apparently almost gangrenous and perhaps covered with a slimy-like coating. I have, on a few occasions, seen vesicles form about the mucous membrane of the lips and gums, but at the stage where I have usually seen them I cannot say that the formation of distinct vesicles is a prominent symptom. Saliva dribbles from the mouth and collects on the lips. Salivation is so extensive often as to form a pool on the ground or floor. The mouth is held tightly shut except that at short intervals the lips or jaws separate very slightly and cause a peculiar but almost diagnostic sucking or smacking noise. In a few aggravated cases I have found the tongue and even the cheeks affected and swollen, but in the great majority of cases the dental pad only. The affection sometimes extends also to the mucous membrane and surrounding tissues about the incisors, but only in a limited number of subjects. The eyes are dull and watery. Saliva is thick and stringy.

Usually we can notice at least some soreness and stiffness in the animal's movements, and often there is marked soreness on pressure about the coronet, and sometimes for several inches up from the foot the cutaneous structures are hot, sore, and swollen. In this first outbreak which came under my notice I saw numerous cases where vesicles formed in this region and several cases where the interdigital area became cracked and sore. These latter cases required three or perhaps four weeks to fully recover.

My second contact with the disease was probably in 1898, when several cases came under my notice, but while it appeared in several different herds often widely separated, yet it was not to be compared in extent to the previous one noted. In October, 1901, I was called to see a few animals on different farms, and consulted about many more presenting the same symptoms.

As already intimated, I have tried to find some authority which would aid in fixing the exact cause of this trouble, but with little success. In speaking of eczema contagiosa, Williams says: "Some observers have thought that the contagium was contained or existed in the form of a vegetable parasite or fungus." This seemed to be about the only support I could get to the conclusions which I had been forced by observation to form, for in these outbreaks I had noticed that the trouble followed a wet spring or early summer, and usually, if not always, after the weather had become hot and comparatively dry, but where grass was still quite plentiful and where a part of the grazing land was low and had been overflowed. But there seem to be peculiar conditions essential to the development of the specific cause. All the animals visited were fed entirely or almost entirely upon blue-grass pastures with perhaps, in a few instances, the addition of some grain feed. Until October 9, 1901, I had never seen a case develop that was traceable to any other feed. These two cases developed almost simultaneously, or but three or four days apart, among a herd of about forty dairy cows. A few of these cows, including these two, had gained access to cow peas in a green state, where they had apparently found the infection. The two cows affected were grade shorthorns in good condition, and both giving milk. One was quite sore in getting about on her feet and extremely sore about the incisor pad and mouth. The other, a younger cow, was only affected about the mouth. The former died within thirty-six hours after I saw her, much to my surprise, as I had never known any other case to terminate fatally. I did not learn of this death for several days afterward, and consequently had no opportunity for examination post-mortem.

I have never noticed the appearance of any vesicles about the anus or vulva or upon the udder. The taking of food when the mouth symptoms are prominent is suspended in almost every instance. The appetite is impaired, perhaps, but not gone, and where the digital symptoms only develop the appetite remains usually good. The animal, of course, loses flesh rapidly, if unable to eat, and becomes weak. Also, if a milch cow, the secretions of milk grow markedly less or cease almost entirely, according to the severity of the case.

I have no special treatment to offer in the way of medication. If possible to do so, I advise removing the cattle for a time from the pasture where infection seems to be, and keeping the affected ones off in a dry lot or barn. Usually I administer Epsom salts to open up the bowels freely, and often prescribe nitrate of potassium two or three times a day for a few days. I use a borax or alum wash for the mouth, to cool and soothe the mucous membrane, and white lotion to the digits. But I consider fully as important, if not the most important feature, is to attend to getting a little food down the animal to keep up strength and prevent as far as may be the extreme loss of flesh. Usually the incisor pad is so sore that the animal cannot even pick up a nubbin of corn when placed before it, and even ground feed or a mash is so irritating when first coming in contact with the diseased mucous membrane as to make the poor brute give up trying. Indeed, she will resist forcing the mouth open at first, but if drenched with gruel, or, what I have found equally as good, take a nubbin of corn, for instance, and place it far back in her mouth, and with the first or second attempt she will learn that she can eat without much pain. In the same way corn blades, cut-grass, etc., may be inserted well back between the molars, and she will usually eat it with a relish.

The recent outbreak of eczema contagiosa in the New England States and the bulletins sent out by the Bureau of Animal Industry under the direction of Dr. Salmon have suggested the similarity of the published clinical symptoms and those of the kind described in the foregoing cases. In each the mouth and feet seem to be the usual seat of attack. In each, one or more of the prominent symptoms may be found without the others being present. The loss is identical in each so far as it goes, *i. e.*, in loss of flesh and loss of milk. A few points, at least, seem to establish the fact that the origin is entirely dissimilar—one is very contagious and attacks various kinds of animals, while I have never seen or heard of a case of the disease in question in anything but the bovine species unless it be slight traces of infection in man in one or two instances. It seems to only become prevalent by exposure to the same cause, which is not contagium, but apparently due to a vegetable fungus. Might not this disease, therefore, be easily confused with the eczema contagiosa and be the cause possibly of the difference of opinion among our older authorities and be the real basis for the opinion merely referred to by

Williams, and previously quoted, but to which he gives no credence. If both diseases were to occur simultaneously, differentiation would be difficult. The occurrence in so many comparatively isolated cases of this symptomatic foot-and-mouth disease strengthens the idea that it is caused by taking into the mouth or stomach some fungus developed through some peculiar climatic and atmospheric conditions and producing an aphthous condition. It is not uncommon for one or a very few cases to be fully developed and no more appear, while often but few, if any, will escape entirely. I have never seen it occur after heavy frosts come, nor in the early spring.

